# Decreased 5‐HT_1A_ binding in mild Alzheimer's disease—A positron emission tomography study

**DOI:** 10.1002/syn.22235

**Published:** 2022-05-28

**Authors:** Patrik Mattsson, Zsolt Cselényi, Bengt Andrée, Jacqueline Borg, Sangram Nag, Christer Halldin, Lars Farde

**Affiliations:** ^1^ Department of Clinical Neuroscience Center for Psychiatry Research, Karolinska Institutet and Stockholm County Stockholm Sweden; ^2^ Personalized Medicine, R&D PET Science Centre, AstraZeneca Stockholm Sweden

**Keywords:** 5‐HT_1A_ receptor, activities of daily living, Alzheimer's disease, cognitive function, neurodegeneration, positron emission tomography

## Abstract

Decreased 5‐HT_1A_ receptor binding has been associated with Alzheimer's disease (AD) and interpreted as a consequence of neuron loss. The purpose of the present study was to compare [^11^C]WAY100635 binding to the 5‐HT_1A_ receptor in the hippocampus, entorhinal cortex, amygdala and pericalcarine cortex in mild AD patients and elderly controls. AD patients (*n* = 7) and elderly control subjects (*n* = 8) were examined with positron emission tomography (PET) and [^11^C]WAY100635. PET data acquisition was performed with an ECAT EXACT HR system. Wavelet‐aided parametric images of nondisplaceable binding potential (*BP*_ND_) were generated using Logan's graphical analysis with cerebellum as the reference region. Correction for partial volume effects was performed with the Müller–Gärtner method. Regions of interest (ROIs) were applied to the individual parametric images, and the regional *BP*_ND_ was calculated as the average parametric voxel value within each ROI. In addition to comparisons between subject groups, correlations between *BP*_ND_ values and scores on the Mini‐Mental State Examination, Disability Assessment for Dementia (DAD), and Neuropsychiatric Inventory were expressed by Pearson correlation coefficients. Mean regional *BP*_ND_ was lower in AD patients than in control subjects, and the difference was statistically significant for the hippocampus, entorhinal cortex, and amygdala. A statistically significant correlation was obtained between hippocampal *BP*_ND_ values and DAD scores. The results of the present study corroborate and extend previous findings of decreased 5‐HT_1A_ binding in AD and strengthen the support for 5‐HT_1A_ receptor PET as a tool for the assessment of neurodegenerative changes in mild AD.

## Graphical abstract

[^11^C]WAY100635 binding was measured with positron emission tomography (PET) in mild Alzheimer's disease (AD) patients and elderly control subjects. The mean nondisplaceable binding potential was lower in the hippocampus, entorhinal cortex, and amygdala in AD patients than in controls, supporting 5‐HT_1A_ receptor PET as a tool for the assessment of early neurodegenerative changes in AD.

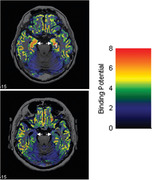

## 1 INTRODUCTION

Alzheimer's disease (AD) is a neurodegenerative disorder characterized by gradual cognitive decline and behavioral changes leading to functional impairment and dementia. The sequence of events leading to the clinical syndrome of AD involves the accumulation of beta‐amyloid (Aβ) oligomers primarily in limbic and association cortices, followed by the gradual deposition of Aβ in plaques. This event is followed by the accumulation of hyperphosphorylated tau in neurofibrillary tangles, widespread neuronal/synaptic dysfunction, and neuronal loss with concomitant neurotransmitter deficits (Selkoe & Hardy, 2016).

The association between these pathogenic events and the clinical syndrome of AD is, however, not fully understood (Gómez‐Isla et al., 1997; Jack et al., 2014; Kril et al., 2002; Nelson et al., 2012). In vivo measurements of Aβ and tau with positron emission tomography (PET) have shown that a considerable proportion of cognitively unimpaired elderly subjects are Aβ‐ and tau‐positive (Pike et al., 2007; Wang & Edison, 2019). While these challenging observations have been interpreted as evidence of preclinical AD, neurodegenerative changes such as synapse and neuron loss have been more closely linked to cognitive impairment and clinical dementia (Andrade‐Moraes et al., 2013; Terry et al., 1991). A substantial fraction of variance in clinical severity is, nevertheless, left unexplained using established biomarkers (Bejanin et al., 2017; Giannakopoulos et al., 2009).

PET imaging of neurotransmitter systems represents another approach to study neurodegeneration in AD (Mecca, 2019). Here, the serotonin 5‐HT_1A_ receptor is of particular interest since it is widely expressed in the brain with particularly high density in the hippocampus and entorhinal cortex (Hall et al., 1997), regions that are vulnerable to pathological change and neuron loss early in AD (Braak et al., 2006; Fu et al., 2018). Decreased 5‐HT_1A_ receptor binding in neocortical and limbic regions, such as the hippocampus and amygdala, has been reported in several postmortem studies in AD and interpreted as a consequence of pyramidal neuron loss (Bowen et al., 1983, 1989; Cross et al., 1984; Crow et al., 1984; Lai et al., 2011; Mizukami et al., 2011; Vidal et al., 2016). Initial PET studies have largely confirmed these findings in vivo and demonstrated decreased 5‐HT_1A_ binding in the hippocampus and medial temporal cortex in AD patients, compared to control subjects (Kepe et al., 2006; Lanctôt et al., 2007; Truchot et al., 2007). In addition, limbic regions have been further explored in a more recent study reporting a decrease in [^18^F]MPPF binding to the 5‐HT_1A_ receptor in the parahippocampus together with a nonsignificant decrease in the amygdala (Truchot et al., 2008).

The primary aim of the present PET study was to confirm previous results of decreased 5‐HT_1A_ binding in AD patients and to further examine 5‐HT_1A_ binding in subregions of the temporal lobe that have been implicated in the early neurodegenerative process of AD (Dubois et al., 2007). We hypothesized that [^11^C]WAY100635 binding to 5‐HT_1A_ would be lower in AD patients, compared to control subjects in the hippocampus, entorhinal cortex, and amygdala. The pericalcarine cortex, a region known to be affected in later stages of AD, was included in the analysis for comparative purposes. Last, we explored correlations between [^11^C]WAY100635 binding and cognitive function, activities of daily living (ADL), and neuropsychiatric symptoms.

## 2 MATERIALS AND METHODS

### 2.1 Subjects

The study was approved by the Regional Ethics Committee of Stockholm and the Radiation Safety Committee at the Karolinska University Hospital. It was performed in accordance with the Declaration of Helsinki and the International Conference on Harmonization/Good Clinical Practice guidelines. All subjects gave written informed consent before participation in the study.

AD patients were recruited from the Memory unit at the Geriatric clinic of Dalens Hospital in Stockholm. The patients were diagnosed shortly before inclusion in the study, and they fulfilled diagnostic criteria for dementia according to diagnostic and statistical manual of mental disorders, 4th ed. (DSM‐IV) and probable AD according to National Institute of Neurological and Communicative Disorders and Stroke and the Alzheimer’s Disease and Related Disorders Association (NINCDS‐ADRDA) criteria (McKhann et al., 2011). The patients were required to have a relative or other close person who could accompany them to the visits and act as an informant for the clinical assessments.

Elderly control subjects were recruited via local advertisements at the Karolinska University Hospital and at meeting points for older people in the community. The control subjects were living without the need for care, had no history of cognitive impairment, and had Mini‐Mental State Examination (MMSE) scores within the normal range (Folstein et al., 1975). The exclusion criteria for AD patients and control subjects were: (1) history of major psychiatric disorder such as schizophrenia, major depression, or bipolar disorder; (2) substance abuse during the last 12 months; (3) medication with serotonergic drugs; and (4) significant metabolic or cerebrovascular disorder.

### 2.2 Clinical characterization

Global cognitive function was assessed with the MMSE. The Clinical Dementia Rating (CDR), a dementia staging instrument based on a semistructured interview of the patient and a reliable informant, was used for staging of clinical severity (Morris, 1993). Functional ability was assessed with the Disability Assessment for Dementia (DAD) scale (Gélinas et al., 1999). The DAD covers 40 items of basic and instrumental ADL (IADL). High scores represent fewer disabilities in ADL, and low scores indicate increased disabilities in ADL. To differentiate between basic ADLs (BADLs), which are maintained until later stages of AD, and IADLs, which are compromised early in AD, the DAD subscale scores covering BADL and IADL were calculated separately (Gélinas et al., 1999). Moreover, the Neuropsychiatric Inventory (NPI; Cummings et al., 1994) was used to assess the frequency and severity of neuropsychiatric symptoms (delusions, hallucinations, agitation, dysphoria, anxiety, apathy, irritability, euphoria, disinhibition, aberrant motor behavior, night‐time behavior disturbances, and appetite and eating abnormalities) in the AD patients.

### 2.3 MRI

Magnetic resonance imaging (MRI) was performed using the 1.5T GE Signa system (General Electric Healthcare) at the MRI center of the Karolinska University Hospital. To rule out pathology not consistent with a diagnosis of AD, the MRI images were evaluated by a neuroradiologist. The T1‐weighted spoiled gradient recalled echo (SPGR) images were used as an anatomical reference for the delineation of regions of interest (ROIs).

### 2.4 PET experimental procedure

An individual plaster helmet was made for each subject prior to PET and used during the PET measurement to minimize head movements (Bergström et al., 1981). Radiosynthesis of [^11^C]WAY100635 was performed according to a procedure described previously (Hall et al., 1997). The mean radioactivity injected intravenous (i.v.) and the mean molar activity was 249 (232–272) MBq and 51 (20–120) GBq/μmol, respectively, for the controls. The corresponding values for the AD patients were 256 (242–273) MBq and 37 (19–65) GBq/μmol. The molar activity could not be obtained in one of the AD patients since an insufficient amount of product was left for analysis after i.v. injection.

PET data acquisition was performed with the ECAT EXACT HR 47 system (Siemens Medical Solutions). The transaxial spatial resolution is 3.6 mm full width at half maximum (FWHM) at the center of the field of view and 4.0 mm (FWHM) axially (Wienhard et al., 1994). Following i.v. bolus injection of [^11^C]WAY100635, brain radioactivity was measured for 69 min in a series of 16 frames (3 × 1, 4 × 3, 9 × 6 min).

### 2.5 PET image analysis

MRI images were coregistered to the individual PET images using SPM5 (Wellcome Department of Imaging Neuroscience). The selection of ROIs was hypothesis‐driven and based on the literature describing regions with early pathology in the medial temporal lobe and regions with late pathology in the occipital lobe (Braak et al., 2006; Dubois et al., 2007; Gómez‐Isla et al., 1996; Price et al., 2001) as well as previous PET studies reporting findings in the medial temporal cortex and hippocampus (Kepe et al., 2006; Lanctôt et al., 2007; Truchot et al., 2008, 2007). ROIs were defined for the hippocampus, entorhinal cortex, amygdala, and pericalcarine cortex using FreeSurfer (version 5.0.0, http://surfer.nmr.mgh.harvard.edu/). The cerebellar cortex was used as a reference region for free and nonspecifically bound [^11^C]WAY100635 in the brain (Farde et al., 1998). The cerebellar cortex was trimmed, using only voxels above the lowest plane of the pons, behind and at/below the posterior tip of the fourth ventricle. Correction for partial volume effects (PVE) was performed with the Müller‐Gärtner (MG) method (Müller‐Gärtner et al., 1992).

### 2.6 Quantification

Quantification was performed using wavelet aided parametric imaging (WAPI), which employs the reference region‐based Logan graphical analysis to estimate the binding potential (*BP*_ND_) for each voxel (Cselényi et al., 2002, 2006). The background and procedure for the wavelet‐based analysis have previously been described in detail (Cselényi et al., 2002, 2006). In summary, the original images were transformed frame‐by‐frame to the wavelet space. The depth of decomposition was 2, and the length of the filter kernels was 22 (defined previously as the best trade‐off between computational efficiency and output quality). ROIs were applied to the individual parametric images, and the regional nondisplaceable binding potential (*BP*_ND_) was calculated as the average voxel value inside each ROI.

### 2.7 Statistical analysis

Statistical analysis was performed with R version 3.4.3 (R Core Team (2017; R: A language and environment for statistical computing R Foundation for Statistical Computing, https://www.R‐project.org/). Group differences in *BP*_ND_ and ROI volumes were analyzed with independent *t*‐tests. Effect sizes were obtained by calculating Cohen's *d*. Associations between *BP*_ND_ and clinical variables were examined by calculating Pearson correlation coefficients (*r*). Semipartial correlations controlling for the effect of MMSE on DAD scores were explored since cognitive function has been shown to predict ADL in mild AD (Liu‐Seifert et al., 2015). Bonferroni correction for multiple comparisons was applied in the correlation analysis. *p* ≤ .05 was considered statistically significant.

## 3 RESULTS

### 3.1 Demographics and clinical characteristics

Seven patients with AD and eight controls were included in the study and participated according to the protocol. Demographic data and clinical characteristics are summarized in Table 1. There were no significant age or sex differences between the AD group and the control group. The mean MMSE score was significantly lower in the AD group than in the control group. The CDR score was .5 (very mild dementia) in one AD patient, and in the remaining AD patients, the CDR score was 1 (mild dementia). The mean DAD score of 83.4 corresponds to an approximately 17% mean loss of functional ability in the AD sample. At a more detailed level, the mean DAD subscale score of BADL was 93, whereas the mean IADL score was 74. Neuropsychiatric symptoms of mainly mild severity were present in five of the seven AD patients (71%) and included symptoms of dysphoria, anxiety, apathy, agitation, irritability, sleep disturbance, appetite disturbance, delusions, and hallucinations.

**TABLE 1:** Demographic data and clinical characteristics

	AD patients (*n* = 7)	Controls (*n* = 8)	*p*‐value
Age	75.9 (65–80)	73.4 (66–78)	.33^a^
Sex (F/M)	5/2	5/3	.71^b^
MMSE	23.9 (19–29)	29.9 (29–30)	.0001^a^
CDR	0.5–1	–	–
DAD	83.4 (69–100)	–	–
BADL	93.0 (79–100)		
IADL	74.0 (53–100)		
NPI	10.9 (0–26)	–	–

*Note*: Values are the mean (range), except for sex (F = female/M = male) and

CDR (categorical).

Abbreviations: AD, Alzheimer's disease; BADL, basic activities of daily living subscale; CDR, Clinical Dementia Rating; DAD, Disability Assessment for Dementia; IADL, instrumental activities of daily living subscale; MMSE, Mini‐Mental State Examination; NPI, Neuropsychiatric Inventory.

^a^ *t* test;

^b^ *X^2^*‐test.

### 3.2 [^11^C]WAY100635 binding to the 5‐HT_1A_ receptor

**FIGURE 1:**
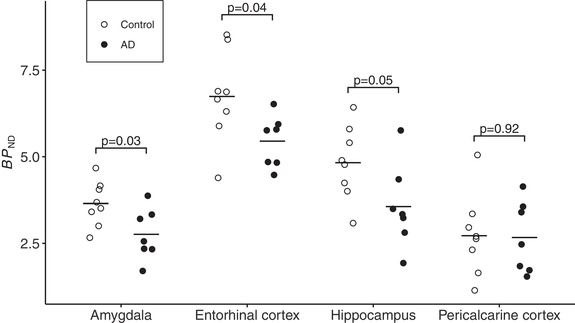
Individual regional nondisplaceable binding potential (*BP*_ND_) values (horizontal lines indicate mean values) for control subjects (○) and Alzheimer's disease (AD) patients (●)

All subjects participated in one PET examination with [^11^C]WAY100635. In all examined brain regions, the mean *BP*_ND_ was numerically lower in AD patients than in control subjects. The difference was statistically significant for the hippocampus, entorhinal cortex, and amygdala (Figure 1, Table 2), and the effect sizes were large as estimated with Cohen's *d* (*d* = 1.12, 95% confidence interval (CI) = .03, 2.21 for the hippocampus; *d* = 1.18, 95% CI = .08, 2.27 for the entorhinal cortex; *d* = 1.28, 95% CI = 1.17, 2.40 for the amygdala). The difference in mean *BP*_ND_ in the pericalcarine cortex was small and nonsignificant. The ROI volumes are also presented in Table 2. Group differences in volume were statistically significant for the hippocampus and amygdala but of smaller magnitude than group differences in partial volume corrected *BP*_ND_.

**TABLE 2:** Regional [^11^C]WAY100635 *BP*_ND_ values and volumes

	*BP*_ND_						Volume (cm^3^)					
	**Controls**		**AD patients**				**Controls**		**AD patients**			
ROI	***M***	***SD***	***M***	***SD***	***p***	**Difference**	***M***	***SD***	***M***	***SD***	***p***	**Difference**
Amygdala	3.65	.65	2.76	.74	.03	−24%	2.88	.20	2.39	.42	.01	−17%
Hippocampus	4.83	1.06	3.56	1.21	.05	−26%	8.14	.81	6.93	.92	.02	−15%
Entorhinal cortex	6.74	1.33	5.45	.74	.04	−19%	2.94	.58	2.64	.72	.38	−10%
Pericalcarine cortex	2.72	1.18	2.67	1.03	.92	−2%	3.91	.46	3.80	1.19	.81	−3%

Abbreviations: AD, Alzheimer's disease; *BP*_ND_, nondisplaceable binding potential; ROI, region of interest.

### 3.3 Correlations between regional [^11^C]WAY100635 binding and global cognitive function

No statistically significant correlations were obtained between [^11^C]WAY100635 *BP*_ND_ and MMSE scores, either when the combined sample of control subjects and AD patients was analyzed or when the analysis was restricted to the AD patients.

### 3.4 Correlations between [^11^C]WAY100635 binding and ADL

**FIGURE 2:**
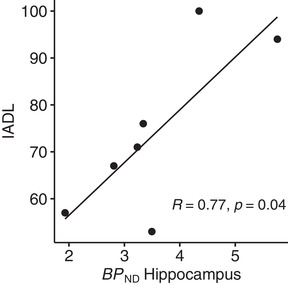
Instrumental activities of daily living scores plotted against *BP*_ND_ values for the hippocampus in AD patients (●)

Positive correlations were obtained between [^11^C]WAY100635 *BP*_ND_ in the hippocampus and DAD total and subscale scores. The correlations were at a trend level for total DAD (*r* = .74, *p* = .057), statistically significant for IADL (*r* = .77, *p* = .042; Figure 2), and nonsignificant for BADL (*r* = .31, *p* = .505). However, the correlation between [^11^C]WAY100635 *BP*_ND_ in the hippocampus and IADL did not remain statistically significant after Bonferroni correction for multiple comparisons, that is, four regions. When controlling for the effect of MMSE on DAD scores by semipartial correlations, statistically significant positive correlations were obtained between [^11^C]WAY100635 *BP*_ND_ in the hippocampus and total DAD scores (*r* = .92, *p* = .010) and IADL scores (*r* = .98, *p* = .00049), whereas the correlation with BADL remained unchanged (*r* = .32, *p* = .538). No statistically significant correlations or semipartial correlations were obtained between [^11^C]WAY100635 *BP*_ND_ and DAD total and subscale scores in any of the other brain regions. The semipartial correlation between [^11^C]WAY100635 *BP*_ND_ in the hippocampus and IADL scores remained statistically significant after correction for multiple comparisons.

### 3.5 Correlations between [^11^C]WAY100635 binding and neuropsychiatric symptoms

No statistically significant correlations were obtained between regional [^11^C]WAY100635 *BP*_ND_ and NPI scores.

## 4 DISCUSSION

In the present PET study, we confirmed the hypothesis of lower [^11^C]WAY100635 binding in the hippocampus, entorhinal cortex, and amygdala in AD patients than in control subjects. The results corroborate and extend the results from previous PET studies reporting lower [^18^F]MPPF binding in the hippocampus and lower [^11^C]WAY100635 binding in the medial temporal cortex of AD patients than in controls (Kepe et al., 2006; Lanctôt et al., 2007; Truchot et al., 2007). Whereas two of the previous studies included patients with moderate and severe AD (Kepe et al., 2006; Lanctôt et al., 2007), our results in very mild ‐ mild AD patients strengthen the evidence of decreased in vivo 5‐HT_1A_ binding already in early AD (Truchot et al., 2007). In addition, the changes in 5‐HT_1A_ binding do not seem to be global in early AD as demonstrated by the almost identical mean *BP*_ND_ in the pericalcarine cortex.

Reduced binding potential, as measured with PET, can be interpreted as a reduced number of receptor proteins in the nerve terminals, reduced arborization of the dendritic tree, or a reduced number of neurons. The resolution of PET is, however, not sufficient to differentiate between these three interpretations. Decreased dendritic spine density is prominent early in AD and correlates with synapse loss and cognitive decline (Baloyannis, 2009; Dorostkar et al., 2015; Scheff et al., 2007). Neuron loss is also prominent in the medial temporal lobe in very mild AD and is suggested to distinguish patients with dementia from nondemented aging (Gómez‐Isla et al., 1996; Price et al., 2001). The localization of the 5‐HT_1A_ receptor on neuronal cell bodies, dendrites and synapses (Mengod et al., 2010) suggests that decreased [^11^C]WAY100635 binding reflects both synapse and neuron loss. Furthermore, postmortem studies have concluded that decreased 5‐HT_1A_ binding may be restricted to advanced stages of AD (Crow et al., 1984; Mizukami et al., 2011; Vidal et al., 2016). Our finding of decreased 5‐HT_1A_ binding in mild AD, however, suggests that early neurodegenerative changes, with synapse and neuron loss in the medial temporal lobe, can also be detected with PET and [^11^C]WAY100635.

The decreased [^11^C]WAY100635 binding to 5‐HT_1A_ receptors in the amygdala warrants further comment. After the entorhinal cortex and the hippocampus, the amygdala is one of the first structures to become affected by AD neuropathology (Braak et al., 2006). Moreover, a few early postmortem studies showing severe neuron loss in the amygdala have been performed in advanced AD cases, and the results suggest that neuron loss in the amygdala could be greater than in any other region of the brain (Herzog & Kemper, 1980; Hopper & Vogel, 1976; Scott et al., 1992; Vereecken et al., 1994). More recent MRI studies in AD have shown early and prominent atrophy of the amygdala, at the same level or even exceeding the level of hippocampal atrophy (Klein‐Koerkamp et al., 2014; Poulin et al., 2011). Although postmortem correlates of these atrophic changes have not been reported in mild AD, our finding of decreased 5‐HT_1A_ binding in the amygdala in mild AD patients could reflect early neuron loss at a level that is similar to neuron loss in the hippocampus and entorhinal cortex.

We did not obtain any significant correlations between [^11^C]WAY100635 binding and MMSE scores in our sample of very mild‐mild AD patients. Positive correlations between 5‐HT_1A_ binding and global cognitive function have been reported in previous PET studies that included AD patients with a wider range of disease severity and that combined samples of AD patients and controls in the analysis (Kepe et al., 2006; Lanctôt et al., 2007). Our results are, on the other hand, in line with a previous PET study of 5‐HT_1A_ binding in mild AD, where no associations with cognitive function in AD were observed (Truchot et al., 2007). However, when exploring correlations between 5‐HT_1A_ binding and ADL in the group of AD patients, we obtained a significant positive correlation (nonsignificant after correction for multiple comparisons) between [^11^C]WAY100635 and IADL. After controlling for the effect of MMSE on ADL (Liu‐Seifert et al., 2015), both global DAD scores and IADL scores were strongly correlated with 5‐HT_1A_ binding in the hippocampus, and the correlation with IADL remained statistically significant after correction for multiple comparisons. The observed association could be a reflection of neurodegenerative changes (Jutten et al., 2019) but may also point to a functional relevance of the 5‐HT_1A_ receptor in AD.

Studies of serotonergic correlates of functional impairment in AD are scarce, and clinical trials of selective serotonin reuptake inhibitors (SSRIs) that included ADL as a secondary outcome have not shown any significant benefit on ADL (Jones et al., 2016). In one previous clinical trial that specifically addressed this question, serotonin augmentation with an SSRI in nondepressed AD patients was associated with beneficial effects on ADL (Mowla et al., 2007). However, whether this effect could be mediated by the 5‐HT_1A_ receptor is not known. Since executive function has been shown to explain a larger amount of variance in IADL than global cognitive status (Mcalister et al., 2016), it is interesting to note that treatment with the 5‐HT_1A_ agonist tandospirone improves executive function (Baba et al., 2015; Sumiyoshi et al., 2001). Furthermore, although the executive function is mostly ascribed to the prefrontal cortex, hippocampal neurodegeneration has been associated with executive dysfunction in early AD (Nagata et al., 2011). The correlation between 5‐HT_1A_ binding and IADL in the present study must be interpreted cautiously due to the small sample size and needs to be replicated in a larger study before any firm conclusions can be drawn about a potential association between the serotonin system and function in mild AD.

In summary, the results of the present study corroborate and extend previous reports of decreased 5‐HT_1A_ binding in the hippocampus and medial temporal cortex in AD. In addition, we found support for lower binding in the entorhinal cortex and amygdala in mild AD patients. The changes in 5‐HT_1A_ binding in early AD seem to be regional rather than global since the mean *BP*_ND_ was unchanged in a cortical “control” region. Moreover, an exploratory correlation analysis indicated associations between 5‐HT_1A_ binding and functional impairment. These results add to the growing support for PET imaging of neurotransmitter systems and synaptic density as potential biomarkers for the evaluation of neurodegenerative changes in AD (Bastin et al., 2020; Chen et al., 2018; Mecca et al., 2020).

### 4.1 Limitations

The small sample size is a limitation of the present study. Our results of decreased binding in subregions of the medial temporal lobe are, however, in line with results from previous PET studies that have shown decreased 5‐HT_1A_ receptor binding of similar magnitude in the medial temporal lobe in AD.

Another consideration is the potential influence of atrophy that may lead to lower BP values in the AD group due to PVE. For that purpose, we applied partial volume correction (PVC) according to MG. However, it cannot be excluded that PVC did not fully compensate for volume differences contributing to differences in *BP*_ND,_ and future studies might examine other approaches to PVE correction. Even if the observed results are due to an undetermined combination of reduced receptor density and atrophy, 5‐HT_1A_ imaging still may be more sensitive to early changes in AD than other approaches.

## 5 CONCLUSION

PET‐measured 5‐HT_1A_ receptor binding has potential as a biomarker of neurodegeneration and functional impairment in mild AD.

## CONFLICT OF INTEREST

The authors have no conflicts of interest to declare.

## Data Availability

The data that support the findings of this study are available from the corresponding author on reasonable request.
